# Association of Plasma Angiotensin-(1–7) Level and Left Ventricular Function in Patients with Type 2 Diabetes Mellitus

**DOI:** 10.1371/journal.pone.0062788

**Published:** 2013-05-14

**Authors:** Pan-Pan Hao, Yu-Guo Chen, Yan-Ping Liu, Ming-Xiang Zhang, Jian-Min Yang, Fei Gao, Yun Zhang, Cheng Zhang

**Affiliations:** Key Laboratory of Cardiovascular Remodeling and Function Research, Chinese Ministry of Education and Chinese Ministry of Public Health, Qilu Hospital, Shandong University, Jinan, Shandong, China; Thomas Jefferson University, United States of America

## Abstract

**Background:**

We recently found that overexpression of angiotensin (Ang)-converting enzyme 2, which metabolizes Ang-II to Ang-(1–7) and Ang-I to Ang-(1–9), may prevent diabetes-induced left ventricular remodeling and dysfunction in rats. Our objective was to evaluate the association of plasma Ang-(1–7) level and left ventricular function in patients with type 2 diabetes mellitus.

**Methodology/Principal Findings:**

We measured the left ventricular ejection fraction (EF), ratio of early to late left ventricular filling velocity (E/A) and ratio of early diastolic mitral inflow to annular velocity (E/Ea) by ultrasonography in 110 patients with type 2 diabetes mellitus for more than 5 years. Anthropometric and fasting blood values were obtained from medical records. The plasma Ang-(1-7) level in patients with a poor EF (<50%) was significantly lower than that in patients with EF ≥50%; the level in patients with E/A <1 was significantly lower than that in patients with E/A ≥1; and the level in patients with E/Ea >15 was significantly lower than that in patients with E/Ea ≤15. Ang-(1–7) level was negatively correlated with E/Ea and Log-N-terminal pro-B-type natriuretic peptide and positively with EF and E/A. Stepwise multiple regression analysis revealed that Ang-(1–7), hemoglobin A1c and Ang-II levels as well as duration of diabetes predicted EF; Ang-(1–7) level, fasting blood glucose, low-density lipoprotein cholesterol level and duration of diabetes predicted E/A; and Ang-(1–7) and hemoglobin A1c levels predicted E/Ea.

**Conclusions/Significance:**

Plasma Ang-(1–7) level is independently associated with left ventricular function in patients with type 2 diabetes mellitus and may be a biomarker for assessing cardiac function in such patients.

## Introduction

The incidence of type 2 diabetes mellitus has been increasing worldwide and rapidly assuming epidemic proportions over the last several decades. A number of epidemiological, clinical and autopsy studies have proposed that the development of heart failure due to an ill-defined cardiomyopathy is a distinct clinical entity among diabetic patients, even in the absence of hypertension and coronary artery disease [1]–[4]. Evidence for the occurrence of diabetic cardiomyopathy has been demonstrated in the form of both diastolic and systolic dysfunction and structural abnormalities. Even in asymptomatic diabetic patients, diastolic dysfunction may be common [1]–[4].

An early phase in the development of heart failure is cardiac remodeling, which is thought to be an important aspect of disease progression in heart failure, regardless of cause. Cardiac remodeling is manifested clinically by changes in cardiac size, shape, and function in response to cardiac injury or inflammation [5]. The mechanisms leading to diabetes-induced cardiac remodeling and dysfunction are not completely understood but are thought to arise from a common upstream pathway involving the renin–angiotensin (Ang) system (RAS). The role of this system in cardiac remodeling is exemplified by Ang-converting enzyme (ACE) inhibitors and Ang-II type 1 receptor (AT_1_) blockers, which improve survival in patients with heart failure, slowing down and in some cases even reversing the deregulation of certain variables of cardiac remodeling [6]. Many other mechanisms, such as microvascular disease, autonomic dysfunction, metabolic disorders, and interstitial fibrosis, may cause diabetic cardiomyopathy [7]. However, the exact etio-pathogenesis still remains unclear.

Ang-(1–7) is formed in the heart from Ang-I or -II by several endopeptidases and carboxypeptidases, including ACE2. Recently, we and others reported that both ACE2 and Ang-(1–7) had cardioprotective effects in experimental animals as well as in cultured cardiac fibroblasts and myocytes [8]–[13]. And we further found that plasma Ang-(1–7) level was independently associated with ameliorating cardiac damage and function after reperfusion therapy in patients with acute myocardial infarction [14]. However, the relation between plasma Ang-(1–7) level and cardiac remodeling and function in diabetic patients is not clear.

In this study we aimed to quantify the association of various clinical and other characteristics with left ventricular (LV) dysfunction in patients with type 2 diabetes mellitus.

## Methods

### Ethics Statement

The protocol was approved by the ethics committee of Qilu Hospital of Shandong University, and all patients gave their written informed consent to participate.

### Study Subjects

This study was conducted at Qilu Hospital of Shandong University, Jinan, China. Between February and September 2012, we included 110 consecutive normotensive patients with type 2 diabetes mellitus for more than 5 years with no clinical evidence of cardiac disease. Diabetes was defined by fasting blood glucose (FBG) ≥7 mmol/l or use of glucose-lowering drugs. We excluded patients with evidence of coronary artery disease (history of angina, chest pain, electrocardiography changes and abnormal Treadmill test results); hypertension; valvular disease; end-stage hepatic or renal disease or malignancy; and poor transthoracic echo window.

### Anthropometric and Fasting Blood Variables

Values for anthropometric and fasting blood variables were obtained retrospectively from medical charts. Trained research staff measured height, weight and blood pressure. Blood pressure was based on the average of the last 3 of 7 measurements after patients rested for a few minutes in a sitting position. After a 12-hour fast, a venous blood sample was collected and sent to the biochemistry laboratory for estimating glycated hemoglobin A1c level, FBG, and serum lipid profile, including serum levels of triglyceride (TG), total serum cholesterol (TC), high-density lipoprotein cholesterol (HDL-C), low density lipoprotein cholesterol (LDL-C) and N-terminal pro-B-type natriuretic peptide (NT-proBNP).

Blood was obtained in a cocktail of protease inhibitors and plasma Ang-(1–7) and Ang-II levels were determined by use of 2 commercial ELISA kits (Uscnlife, Wuhan, China) of all samples in duplicate.

### Echocardiography

Included patients underwent resting transthoracic 2D echocardiography to assess LV systolic and diastolic function by use of Philips iE33 ultrasound system (Philips Ultrasound, Bothell, WA) according to the standard protocol. M-mode images were obtained for measuring LV end-systolic volume (LVESV) and LV end-diastolic volume (LVEDV) according to American Society of Echocardiography guidelines [15]. As a measure of systolic function, LV ejection fraction (EF) was calculated as EF (%) = (LVEDV – LVESV)/LVEDV × 100, with EF ≥50% considered normal. Diastolic function was assessed by pulsed-wave Doppler imaging of the transmitral filling pattern with the early transmitral filling wave (E-wave) followed by the late filling wave due to atrial contraction (A-wave). For tissue Doppler imaging, early diastolic peak annular velocity (Ea) was obtained with a 2-mm sample volume placed at the lateral side and septal side of the mitral valve annulus. LV diastolic dysfunction was considered present with E/A ratio <1 or >2 or E/Ea ratio >15 as described [16]. All echocardiographic measurements were averaged over 3 consecutive cardiac cycles by an experienced technician who was blinded to study group.

### Statistical Analysis

All continuous variables are expressed as mean ± SEM or median [interquartile range] unless otherwise stated. Data were analyzed by use of SPSS v19 (SPSS Inc., Chicago, IL). We compared characteristics of patients with EF <50% and ≥50%. Patients were also classified by thresholds of E/A and E/Ea. After testing for normality and equality of variance, continuous variables were compared by unpaired *t* test, Welch’s test or Mann-Whitney U test. To test our hypothesis that Ang-(1–7) level was associated with LV function, we first investigated potential determinants of LV function by univariate analysis with Pearson’s or Spearman’s rank correlation coefficient following testing for normality, then identified variables that predicting cardiac variables by stepwise multiple regression analysis. Variables associated with cardiac variables at *p*<0.10 were entered in multivariate analyses, and when 2 independent variables showed strong collinearity (*γ* >0.70), one was selected. Interactions between any 2 sets of the variables remaining in the stepwise models were assessed by centered cross terms. A 2-sided *p*<0.05 was considered statistically significant for the final models.

## Results

### Characteristics of Study Subjects

The characteristics of the 110 patients by EF<and ≥50% are in Table 1∶28 (25.5%) had systolic dysfunction (EF <50%). Diabetes duration, Ang-II level, E/Ea and NT-proBNP were significantly greater and Ang-(1–7) level and E/A were lower for patients with than without systolic dysfunction. Characteristics of the 2 groups by E/A threshold are in Table 2 and those by E/Ea threshold are in Table 3. Diabetes duration and hemoglobin A1c level were greater, Ang-(1–7) level was lower, and FBG was greater but not significantly in patients with E/A <1 than ≥1. Diabetes duration, hemoglobin A1c level, FBG and Ang-II level were greater and Ang-(1–7) level was lower for patients with E/Ea >15 than ≤15.

**Table 1:** Characteristics of patients by ejection fraction (EF).

	EF <50%	EF ≥50%	*p*-value
No. of subjects, *n* (%)	28 (25.5)	82 (74.6)	
Age, yr	65.71±2.03	66.29±0.79	0.7918^b^
Duration of DM, yr	17.75±0.70	12.50±0.43	**<0.0001**
BMI, kg/m^2^	23.58±0.82	23.93±0.32	0.6971^b^
SBP, mmHg	113.2±2.7	119.0 [106.0–132.0]	0.1841^a^
HbA1c, %	7.16±0.10	6.95 [6.70–7.20]	0.2054^a^
FBG, mmol/l	10.69±0.52	9.50 [8.30–11.73]	0.4903^a^
TG, mg/dl	1.85±0.15	1.60 [1.19–2.27]	0.6261^a^
TC, mg/dl	4.63±0.20	4.71±0.11	0.7372
HDL-C, mg/dl	1.14±0.05	1.14 [0.98–1.29]	0.8155^a^
LDL-C, mg/dl	2.76±0.21	2.84±0.09	0.6903
Ang-(1–7), pg/ml	17.00 [14.10–20.35]	19.77±0.43	**0.0059** a
Ang-II, pg/ml	22.61±0.46	21.38±0.28	**0.0259**
E/A	1.02±0.03	1.21±0.02	**<0.0001** b
E/Ea	17.54±0.49	14.12±0.44	**<0.0001** b
NT-proBNP, pg/ml	847.5 [643.3–1281]	235.0 [111.3–466.3]	**<0.0001** a

Data are number (%), mean±SEM or median [interquartile range].

a Mann-Whitney U test.

b Welch’s test.

DM, diabetes mellitus; BMI, body mass index; SBP, systolic blood pressure; HbA1c, hemoglobin A1c; FBG, fasting blood glucose; TG, triglycerides; TC, total cholesterol; HDL-C, high-density lipoprotein cholesterol; LDL-C, low-density lipoprotein cholesterol; E/A, ratio of early to late left ventricular filling velocity; E/Ea, ratio of early diastolic mitral inflow to annular velocity; NT-proBNP, N-terminal pro-B-type natriuretic peptide.

**Table 2:** Characteristics of patients by ratio of early to late left ventricular filling velocity (E/A).

	E/A <1	E/A ≥1	*p*-value
No. of subjects, *n* (%)	32 (29.1)	78 (70.9)	
Age, yr	65.94±1.59	66.23±0.89	0.8651
Duration of DM, yr	14.84±0.65	12.05±0.44	**0.0007**
BMI, kg/m^2^	23.30±0.51	24.06±0.39	0.2716
SBP, mmHg	115.1±2.6	117.1±1.6	0.4964
HbA1c, %	7.29±0.09	6.90 [6.68–7.20]	**0.0023** a
FBG, mmol/l	11.19±0.52	9.40 [8.40–11.50]	0.0663^a^
TG, mg/dl	1.52 [1.19–2.10]	1.66 [1.19–2.46]	0.5736^a^
TC, mg/dl	4.70 [3.79–5.35]	4.69±0.12	0.8875^a^
HDL-C, mg/dl	1.11±0.03	1.14 [0.96–1.34]	0.4590^a^
LDL-C, mg/dl	2.92±0.13	2.78±0.11	0.4677
Ang-(1–7), pg/ml	16.19±0.49	20.48±0.43	**<0.0001**
Ang-II, pg/ml	22.07±0.38	21.54±0.30	0.2822^b^
EF, %	51.91±2.19	59.71±1.23	**0.0014**
E/Ea	17.80±0.42	13.84±0.44	**<0.0001** b
NT-proBNP, pg/ml	565.5 [353.8–850.3]	269.0 [116.5–643.3]	**0.0016** a

Data are number (%), mean±SEM or median [interquartile range].

a Mann-Whitney U test.

b Welch’s test.

DM, diabetes mellitus; BMI, body mass index; SBP, systolic blood pressure; HbA1c, hemoglobin A1c; FBG, fasting blood glucose; TG, triglycerides; TC, total cholesterol; HDL-C, high-density lipoprotein cholesterol; LDL-C, low-density lipoprotein cholesterol; EF, ejection fraction; E/Ea, ratio of early diastolic mitral inflow to annular velocity; NT-proBNP, N-terminal pro-B-type natriuretic peptide.

**Table 3:** Characteristics of patients by ratio of early diastolic mitral inflow to annular velocity (E/Ea).

	E/Ea >15	E/Ea ≤15	*p*-value
No. of subjects, *n* (%)	54 (49.1)	56 (50.9)	
Age, yr	65.59±1.19	66.68±1.01	0.4881
Duration of DM, yr	13.72±0.52	12.04±0.54	**0.0269**
BMI, kg/m^2^	23.74±0.48	23.94±0.41	0.7521
SBP, mmHg	115.9±1.96	117.5 [106.0–130.5]	0.6667^a^
HbA1c, %	7.20±0.07	6.85 [6.63–7.10]	**0.0037** a
FBG, mmol/l	10.89±0.36	9.30 [8.33–11.28]	**0.0366** a
TG, mg/dl	1.58 [1.05–2.08]	1.79 [1.24–2.60]	0.1288^a^
TC, mg/dl	4.69 [3.82–5.36]	4.68±0.15	0.9905^a^
HDL-C, mg/dl	1.16±0.03	1.14 [0.90–1.32]	0.6952^a^
LDL-C, mg/dl	2.92±0.11	2.72±0.13	0.2621
Ang-(1–7), pg/ml	17.96±0.49	20.46±0.54	**0.0009**
Ang-II, pg/ml	22.36 [21.34–23.89]	20.44 [19.25–23.27]	**0.0199** a
EF, %	52.70±1.51	62.50 [53.00–71.00]	**<0.0001** a
E/A	0.99 [0.91–1.17]	1.28±0.02	**<0.0001** a
NT-proBNP, pg/ml	535.0 [264.0–791.8]	226.0 [95.5–568.0]	**0.0006** a

Data are number (%), mean±SEM or median [interquartile range].

a Mann-Whitney U test.

DM, diabetes mellitus; BMI, body mass index; SBP, systolic blood pressure; HbA1c, hemoglobin A1c; FBG, fasting blood glucose; TG, triglycerides; TC, total cholesterol; HDL-C, high-density lipoprotein cholesterol; LDL-C, low-density lipoprotein cholesterol; EF, ejection fraction; E/A, ratio of early to late left ventricular filling velocity; NT-proBNP, N-terminal pro-B-type natriuretic peptide.

### Variables Correlated with and Predictors of Cardiac Variables

**Figure 1:**
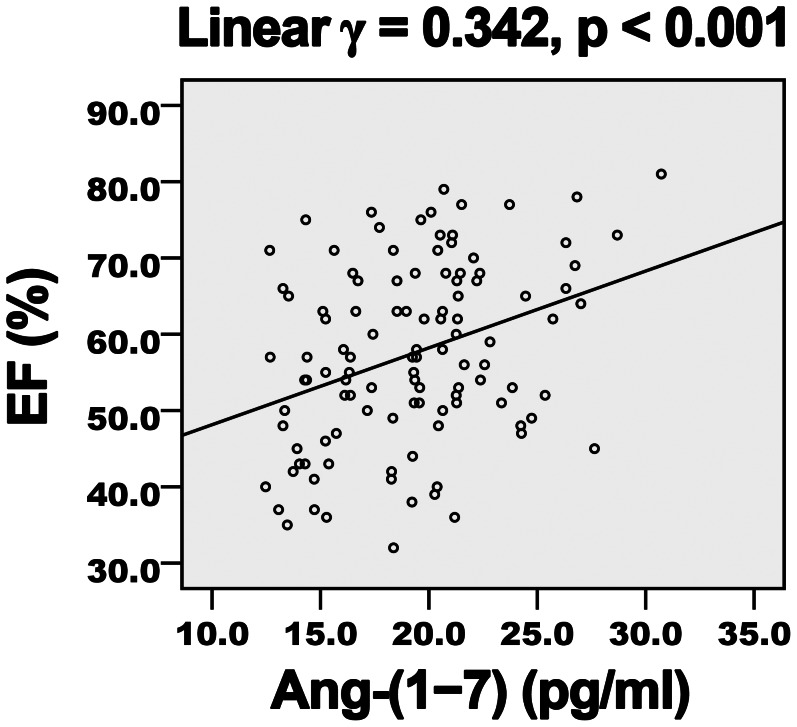
Positive association of plasma Ang-(1–7) level and EF in diabetic patients. EF, ejection fraction; *γ*, Pearson’s correlation coefficient.

**Figure 2:**
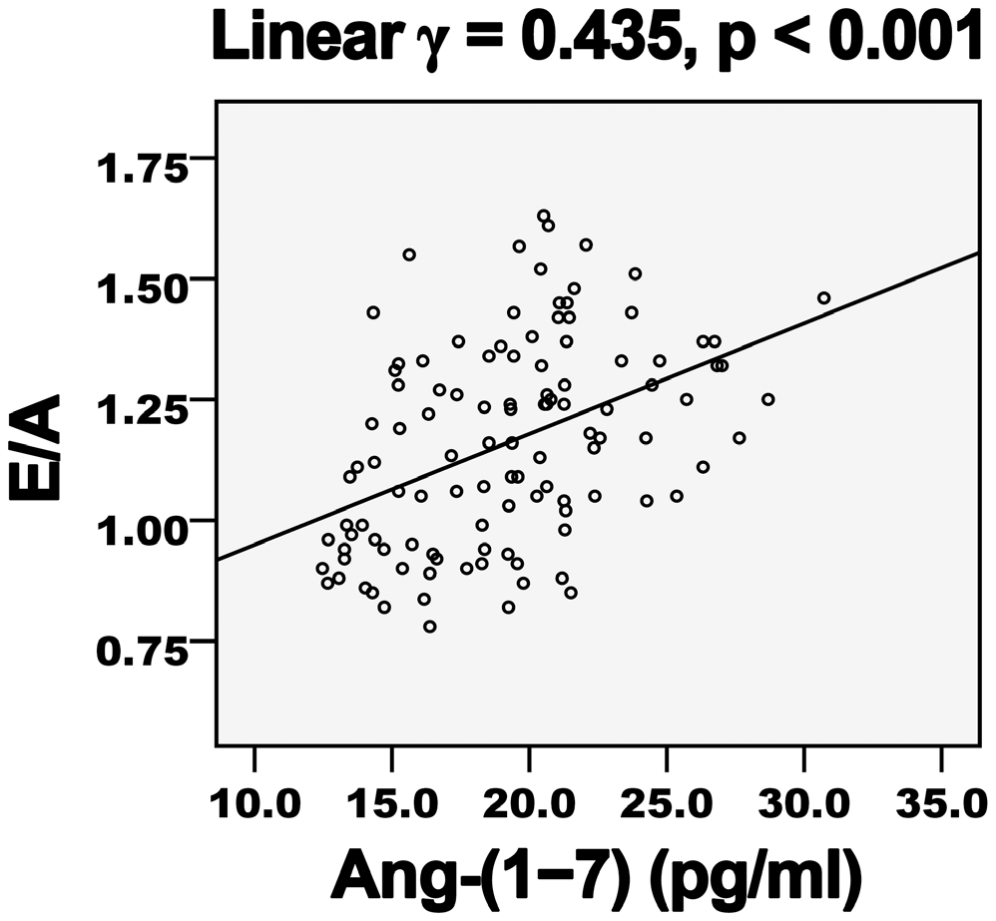
Positive association of plasma Ang-(1–7) level and E/A in diabetic patients. E/A, ratio of early to late LV filling velocity; *γ*, Pearson’s correlation coefficient.

**Figure 3:**
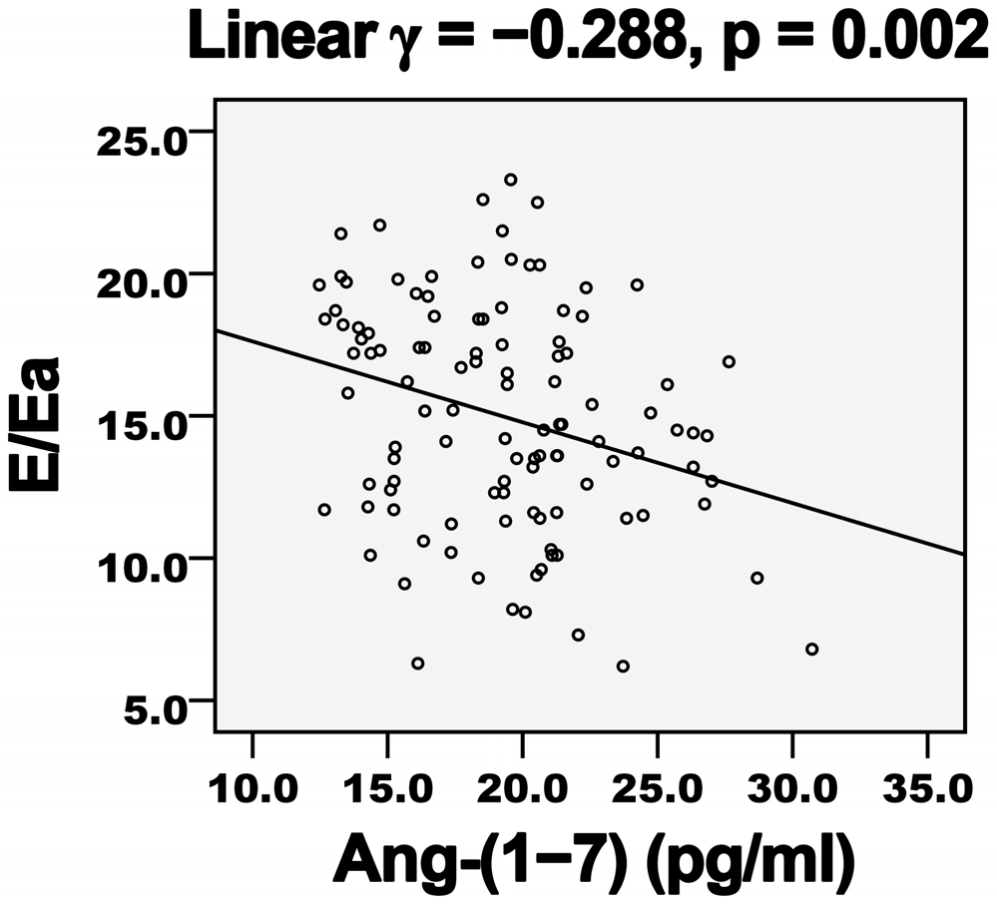
Negative association of plasma Ang-(1–7) level and E/Ea in diabetic patients. E/Ea, ratio of early diastolic mitral inflow to annular velocity; *γ*, Pearson’s correlation coefficient.

**Figure 4:**
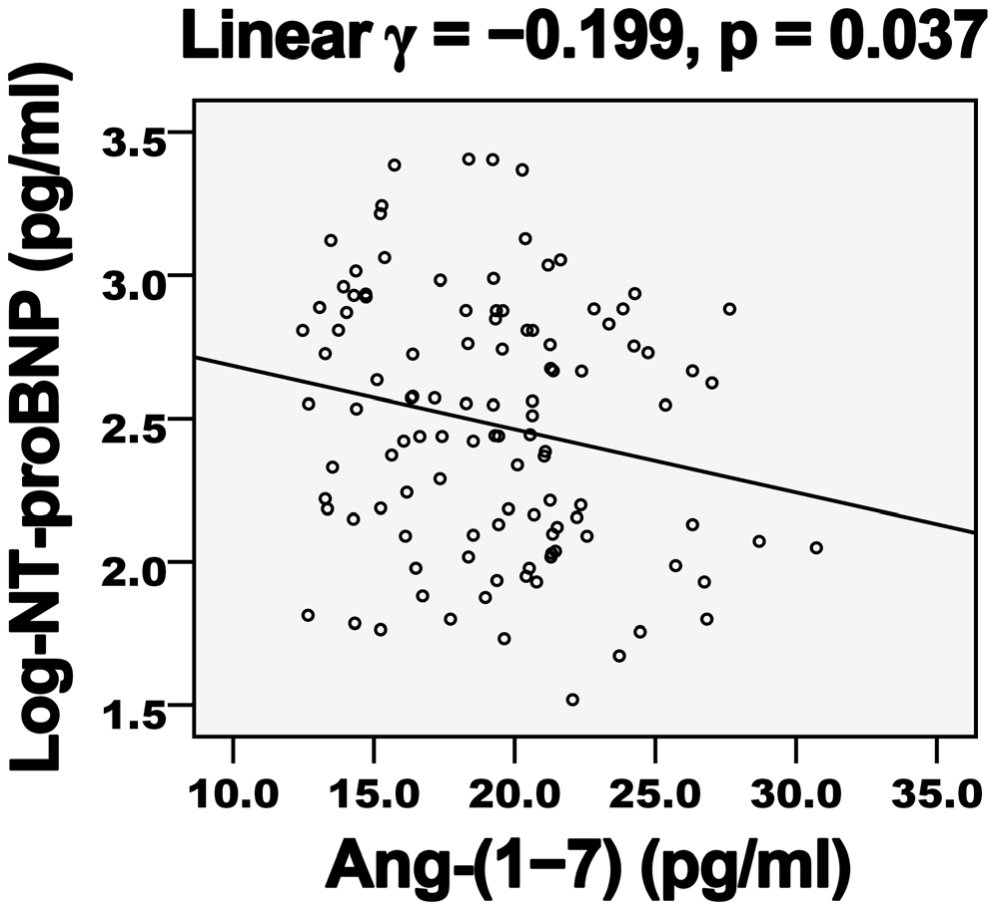
Negative association of plasma Ang-(1–7) level and Log-NT-proBNP in diabetic patients. NT-proBNP, N-terminal pro-B-type natriuretic peptide; *γ*, Pearson’s correlation coefficient.

Diabetes duration, hemoglobin A1c level, FBG and Ang-II level showed a negative correlation (Table 4) and Ang-(1–7) level a positive correlation (Figures 1 and 2) with EF and E/A. LDL-C level showed a negative but non-significant correlation with E/A. Ang-(1–7) level showed a negative correlation (Figures 3 and 4) and diabetes duration, hemoglobin A1c level, FBG and Ang-II level a positive correlation with E/Ea and Log-NT-proBNP. LDL-C showed a positive but non-significant correlation with Log-NT-proBNP.

**Table 4:** Correlation of variables with cardiac function.

	EF	E/A	E/Ea	Log-NT-proBNP
Age	0.038	0.003	−0.083	−0.093
Duration of DM	−0.326**	−0.389***	0.212*	0.233*
BMI	−0.050	0.050	0.056	0.053
SBP	0.083	0.026	−0.106	−0.075
HbA1c^a^	−0.337***	−0.277**	0.261**	0.203*
FBG^a^	−0.222*	−0.225*	0.211*	0.197*
TG^a^	−0.070	0.014	−0.141	0.089
TC	−0.072	−0.104	0.061	0.138
HDL-C^a^	0.133	0.060	0.084	−0.091
LDL-C	−0.112	−0.176^†^	0.146	0.159^†^
Ang-(1–7)	0.342***	0.435***	−0.288**	−0.199*
Ang-II	−0.285**	−0.199*	0.194*	0.300**

a Spearman’s rank correlation coefficients (*ρ*); others are Pearson’s correlation coefficients (*γ*).

* *p*<0.05,

** *p*<0.01,

*** *p*<0.001 and ^†^ *p*<0.1.

EF, ejection fraction; E/A, ratio of early to late left ventricular filling velocity; E/Ea, ratio of early diastolic mitral inflow to annular velocity; NT-proBNP, N-terminal pro-B-type natriuretic peptide; DM, diabetes mellitus; BMI, body mass index; SBP, systolic blood pressure; HbA1c, hemoglobin A1c; FBG, fasting blood glucose; TG, triglycerides; TC, total cholesterol; HDL-C, high-density lipoprotein cholesterol; LDL-C, low-density lipoprotein cholesterol.

All these variables but FBG, which showed strong collinearity with hemoglobin A1c level, were entered into stepwise multiple regression analysis for predicting the cardiac variables. Diabetes duration (*β* = –0.244, *p* = 0.004) and levels of hemoglobin A1c (*β* = –0.223, *p* = 0.009), Ang-(1–7) (*β* = 0.262, *p* = 0.002) and Ang-II (*β* = –0.212, *p* = 0.012) were independent predictors of EF (Table 5A). Diabetes duration (*β* = –0.324, *p*<0.001), hemoglobin A1c level (*β* = –0.211, *p* = 0.010) and Ang-(1–7) level (*β* = 0.365, *p*<0.001) predicted E/A. Levels of hemoglobin A1c (*β* = 0.213, *p* = 0.022) and Ang-(1–7) (*β* = –0.249, *p* = 0.008) predicted E/Ea. Finally, diabetes duration (*β* = 0.199, *p* = 0.030) and Ang-II level (*β* = 0.276, *p* = 0.003) predicted Log-NT-proBNP. Analysis with FBG instead of hemoglobin A1c level shown as model 2 (Table 5B) revealed that diabetes duration, FBG, and LDL-C and Ang-(1–7) levels were predictors of E/A, with a higher coefficient of determination than in model 1. There was no significant interaction between any of 2 sets of the variables in each stepwise model.

**Table 5:** Stepwise linear regression model to predict cardiac function.

(A) Model 1				
	*β*	*p*-value	*R^2^*	Adjusted *R^2^*
EF				
Duration of DM	−0.244	0.004	0.305	0.279
HbA1c level	−0.223	0.009		
Ang-(1–7) level	0.262	0.002		
Ang-II level	−0.212	0.012		
E/A				
Duration of DM	−0.324	<0.001	0.353	0.335
HbA1c level	−0.211	0.010		
Ang-(1–7) level	0.365	<0.001		
E/Ea				
HbA1c level	0.213	0.022	0.127	0.111
Ang-(1–7) level	−0.249	0.008		
Log-NT-proBNP				
Duration of DM	0.199	0.030	0.129	0.113
Ang-II level	0.276	0.003		
**(B) Model 2**				
	***β***	***p*** **-value**	***R^2^***	**Adjusted ** ***R^2^***
EF				
Duration of DM	−0.268	0.002	0.259	0.238
Ang-(1–7) level	0.299	0.001		
Ang-II level	−0.239	0.006		
E/A				
Duration of DM	−0.341	<0.001	0.368	0.344
FBG	−0.186	0.020		
LDL-C level	−0.155	0.048		
Ang-(1–7) level	0.363	<0.001		
E/Ea				
Duration of DM	0.185	0.045	0.117	0.101
Ang-(1–7) level	−0.270	0.004		

*β*, standardized regression coefficient; *R^2^*, coefficient of determination; EF, ejection fraction; DM, diabetes mellitus; HbA1c, hemoglobin A1c; E/A, ratio of early to late left ventricular filling velocity; E/Ea, ratio of early diastolic mitral inflow to annular velocity; NT-proBNP, N-terminal pro-B-type natriuretic peptide; FBG, fasting blood glucose; LDL-C, low-density lipoprotein cholesterol.

## Discussion

Our results reveal a high burden of LV systolic and diastolic dysfunction in patients with type 2 diabetes mellitus and show that plasma Ang-(1–7) level was negatively and significantly associated with E/Ea and Log-NT-proBNP and positively with EF and E/A in diabetic patients. Ang-(1–7) may be a protective mediator and may indicate cardiac remodeling and function in diabetic subjects.

RAS has been suggested to participate in the pathogenesis of diabetic cardiomyopathy. Angiotensinogen, renin, Ang-I and Ang-II are important in diabetic cardiomyopathy. We found high Ang-II level negatively correlated with LV function, as was previously reported [17]. Ang-II is one of the main mediators of diabetes-induced cardiac remodeling that antedates heart failure [18]. Activation of the transforming growth factor β (TGF-β)/Smad pathway may be a sign of high glucose/Ang-II–mediated signaling in the myocardium. Induction of TGF-β1 by high glucose or Ang-II, which then phosphorylates and activates downstream Smad2 and Smad3 proteins, results in cardiac fibrosis, cardiac hypertrophy and heart failure; however, Smad7 can antagonize TGF-β–mediated cardiac remodeling [18]. In addition, mitogen-activated protein kinase (MAPK) signaling is implicated in cardiac diseases and is preferentially activated by high glucose or Ang-II level, thus leading to cardiac fibrosis and dysfunction [19].

ACE inhibitors and AT_1_ blockers are effective in reducing Ang-II–mediated inflammation and end-organ damage in animals with diabetes. During ACE inhibition or AT_1_ blockade, ACE2 activity and mRNA level are elevated in plasma and the left ventricle [20]. We recently reported that ACE2 ameliorated LV remodeling and function in a rat model of diabetic cardiomyopathy, accompanied by downregulated Ang-II and TGF-β1 levels and upregulated Ang-(1–7) expression [8]. The benefits of ACE inhibitors, AT_1_ blockers and ACE2 appear to be mediated in part through Ang-(1–7). Despite lack of evidence supporting the ability of Ang-(1–7) to reduce Ang-II level, Ang-(1–7), which can be generated locally in the myocardium and bind to a distinct plasma membrane G protein-coupled receptor, the Mas receptor, can oppose the effects of Ang-II and has therapeutic effects similar to those of ACE2 on cardiac remodeling in diabetic and other models [10], [12], [21], [22]. Yousif and colleagues recently showed that inhibition of endogenous Ang-(1–7) formation exacerbated diabetes-induced cardiac/renal nitric oxidase activity and end-organ damage [22].

Although we have not established a direct causal link among events, our results show that low endogenous Ang-(1–7) level predicts worse cardiac function in diabetic patients, as indicated by low EF and E/A and high E/Ea and Log-NT-proBNP. Consistent with this result, downregulation of ACE2 was previously found in the late phase of ventricular dysfunction in rats with myocardial infarction [20]. However, in contrast with our results, 2 clinical studies reported that elevated plasma ACE2 activity as a compensatory response was associated with increased severity of myocardial dysfunction and was an independent predictor of adverse clinical events [23], [24]. The reasons for these differences in action between ACE2 and Ang-(1–7) are unclear but could involve elevated ACE activity and ACE/ACE2 ratio in diabetic patients, which accelerates Ang-(1–7) degradation into the inactive fragment Ang-(1–5) [25].

We recently found that plasma Ang-(1–7) levels could predict myocardial salvage after reperfusion treatment for acute myocardial infarction and might indicate long-term myocardial remodeling after acute myocardial infarction [14]. Therefore, it may be not only a biomarker for cardiac function but also a predictor for long-term myocardial remodeling under disease conditions. However, our data showed that plasma Ang-(1–7) level was very low in diabetic patients, especially with cardiac dysfunction, which might be a potential limitation of using Ang-(1–7) as indicative of cardiac function or long-term myocardial remodeling. In animal experiments, the actions of Ang-(1–7) appear to be independent of any blood pressure, FBG or Ang-II lowering effect. Consistent with previous findings, our data indicate that Ang-(1–7) level is not associated with blood pressure, hemoglobin A1c level, FBG, serum lipid profile or even Ang-II level (data not shown). The mechanisms involved in Ang-(1–7)–mediated cardiovascular protection are not well characterized, although the beneficial effects have been reported to be mediated by prostaglandin and nitric oxide [22] and may be related to concerted changes in levels of matrix metalloproteinases and tissue inhibitors of metalloproteinases [10].

ACE2/Ang-(1–7) also have protective effects in the progression of atherosclerosis. We previously reported that ACE2 inhibited and stabilized atherosclerotic plaque via the conversion of Ang-II to vasoprotective Ang-(1–7) [26], [27]. Tesanovic and colleagues recently demonstrated that Ang-(1–7) has both vasoprotection and atheroprotection effects, which may be mediated by the restoration of nitric oxide bioavailability and may involve a complex interaction of both Mas and AT_2_ receptors [28].

Clinical, epidemiological and experimental evidence suggests a ‘female advantage’ in the progression of cardiovascular diseases [29]. However, this ‘gender bias’ may be cancelled by the onset of diabetes in premenopausal women [30]. It is speculated that gender-related differences in sex hormones and intrinsic myocardial and endothelial functions between genders may be responsible for this female “advantage” and “disadvantage” in normal and diabetic conditions. Moreover, recent evidence has suggested that discrepant myocardial Akt activation status may contribute to gender-related differences in myocardial function [31].

There were 2 major limitations in this study. First, it was only a small cross-sectional study and our primary conclusion needs confirmation by additional studies with a large sample size and a prospective design. Second, although the association of plasma Ang-(1–7) level and left ventricular function in patients with type 2 diabetes mellitus was evaluated and confirmed, the differences in Ang-(1–7) levels between subjects with and without diabetes were unclear because normal subjects without diabetes were not enrolled.

In summary, we showed that Ang-(1–7) level could be a biomarker for assessing LV function in diabetic patients because plasma Ang-(1–7) level was independently and negatively correlated with LV remodeling and dysfunction in our diabetic patients. Whether the association of Ang-(1–7) and LV function is attributed to the inhibitory effect of Ang-(1–7) on diabetic myocardial remodeling merits future study. To increase the sample power and verify our results, additional studies with a prospective design and larger cohorts are warranted.

## References

[1] Karvounis HI, Papadopoulos CE, Zaglavara TA, Nouskas IG, Gemitzis KD, et al (2004) Evidence of left ventricular dysfunction in asymptomatic elderly patients with non-insulin-dependent diabetes mellitus. Angiology 55: 549–555. 15378118 10.1177/000331970405500511

[2] Albertini JP, Cohen R, Valensi P, Sachs RN, Charniot JC (2008) B-type natriuretic peptide, a marker of asymptomatic left ventricular dysfunction in type 2 diabetic patients. Diabetes Metab 34: 355–362. 18599336 10.1016/j.diabet.2008.02.004

[3] Shrestha NR, Sharma SK, Karki P, Shrestha NK, Acharya P (2009) Echocardiographic evaluation of diastolic function in asymptomatic type 2 diabetes. JNMA J Nepal Med Assoc 48: 20–23. 19529053

[4] Yu XY, Chen HM, Liang JL, Lin QX, Tan HH, et al (2011) Hyperglycemic myocardial damage is mediated by proinflammatory cytokine: macrophage migration inhibitory factor. PLoS One 6: e16239. 21283592 10.1371/journal.pone.0016239 PMC3026813

[5] Shah AM, Hung CL, Shin SH, Skali H, Verma A, et al (2011) Cardiac structure and function, remodeling, and clinical outcomes among patients with diabetes after myocardial infarction complicated by left ventricular systolic dysfunction, heart failure, or both. Am Heart J 162: 685–691. 21982661 10.1016/j.ahj.2011.07.015

[6] Kuenzli A, Bucher HC, Anand I, Arutiunov G, Kum LC, et al (2010) Meta-analysis of combined therapy with angiotensin receptor antagonists versus ACE inhibitors alone in patients with heart failure. PLoS One 5: e9946. 20376345 10.1371/journal.pone.0009946 PMC2848587

[7] From AM, Scott CG, Chen HH (2009) Changes in diastolic dysfunction in diabetes mellitus over time. Am J Cardiol 103: 1463–1466. 19427447 10.1016/j.amjcard.2009.01.358 PMC2700297

[8] Dong B, Yu QT, Dai HY, Gao YY, Zhou ZL, et al (2012) Angiotensin-converting enzyme-2 overexpression improves left ventricular remodeling and function in a rat model of diabetic cardiomyopathy. J Am Coll Cardiol 59: 739–747. 22340266 10.1016/j.jacc.2011.09.071

[9] Feng Y, Hans C, McIlwain E, Varner KJ, Lazartigues E (2012) Angiotensin-converting enzyme 2 over-expression in the central nervous system reduces angiotensin-II-mediated cardiac hypertrophy. PLoS One 7: e48910. 23155428 10.1371/journal.pone.0048910 PMC3498357

[10] Pei Z, Meng R, Li G, Yan G, Xu C, et al (2010) Angiotensin-(1–7) ameliorates myocardial remodeling and interstitial fibrosis in spontaneous hypertension: role of MMPs/TIMPs. Toxicol Lett 199: 173–181. 20837116 10.1016/j.toxlet.2010.08.021

[11] Johnson JA, West J, Maynard KB, Hemnes AR (2011) ACE2 improves right ventricular function in a pressure overload model. PLoS One 6: e20828. 21695173 10.1371/journal.pone.0020828 PMC3112229

[12] Flores-Muñoz M, Godinho BM, Almalik A, Nicklin SA (2012) Adenoviral delivery of angiotensin-(1–7) or angiotensin-(1–9) inhibits cardiomyocyte hypertrophy via the mas or angiotensin type 2 receptor. PLoS One 7: e45564. 23029101 10.1371/journal.pone.0045564 PMC3447802

[13] McCollum LT, Gallagher PE, Tallant EA (2012) Angiotensin-(1–7) abrogates mitogen-stimulated proliferation of cardiac fibroblasts. Peptides 34: 380–388. 22326709 10.1016/j.peptides.2012.01.020 PMC3326596

[14] Hao PP, Liu YP, Hou GH, Zhang MX, Chen YG, et al.. (2013) Usefulness of angiotensin-(1–7) to predict myocardial salvage after percutaneous coronary intervention in patients with acute myocardial infarction. Int J Cardiol. pii: S0167–5273(13)00266–0. doi: 10.1016/j.ijcard.2013.01.206. 10.1016/j.ijcard.2013.01.206 23473823

[15] Sahn DJ, DeMaria A, Kisslo J, Weyman A (1978) Recommendations regarding quantitation in M-mode echocardiography: results of a survey of echocardiographic measurements. Circulation 58: 1072–1083. 709763 10.1161/01.cir.58.6.1072

[16] Ommen SR, Nishimura RA, Appleton CP, Miller FA, Oh JK, et al (2000) Clinical utility of Doppler echocardiography and tissue Doppler imaging in the estimation of left ventricular filling pressures: A comparative simultaneous Doppler-catheterization study. Circulation 102: 1788–1794. 11023933 10.1161/01.cir.102.15.1788

[17] Serneri GG, Boddi M, Cecioni I, Vanni S, Coppo M, et al (2001) Cardiac angiotensin II formation in the clinical course of heart failure and its relationship with left ventricular function. Circ Res 88: 961–968. 11349007 10.1161/hh0901.089882

[18] Rosenkranz S (2004) TGF-beta1 and angiotensin networking in cardiac remodeling. Cardiovasc Res 63: 423–432. 15276467 10.1016/j.cardiores.2004.04.030

[19] Zhang W, Elimban V, Nijjar MS, Gupta SK, Dhalla NS (2003) Role of mitogen-activated protein kinase in cardiac hypertrophy and heart failure. Exp Clin Cardiol 8: 173–183. 19649217 PMC2719157

[20] Ocaranza MP, Godoy I, Jalil JE, Varas M, Collantes P, et al (2006) Enalapril attenuates downregulation of Angiotensin-converting enzyme 2 in the late phase of ventricular dysfunction in myocardial infarcted rat. Hypertension 48: 572–578. 16908757 10.1161/01.HYP.0000237862.94083.45

[21] Singh K, Singh T, Sharma PL (2011) Beneficial effects of angiotensin (1–7) in diabetic rats with cardiomyopathy. Ther Adv Cardiovasc Dis 5: 159–167. 21558085 10.1177/1753944711409281

[22] Yousif MH, Dhaunsi GS, Makki BM, Qabazard BA, Akhtar S, et al (2012) Characterization of Angiotensin-(1–7) effects on the cardiovascular system in an experimental model of type-1 diabetes. Pharmacol Res 66: 269–275. 22580236 10.1016/j.phrs.2012.05.001

[23] Epelman S, Shrestha K, Troughton RW, Francis GS, Sen S, et al (2009) Soluble angiotensin-converting enzyme 2 in human heart failure: relation with myocardial function and clinical outcomes. J Card Fail 15: 565–571. 19700132 10.1016/j.cardfail.2009.01.014 PMC3179261

[24] Epelman S, Tang WH, Chen SY, Van Lente F, Francis GS, et al (2008) Detection of soluble angiotensin-converting enzyme 2 in heart failure: insights into the endogenous counter-regulatory pathway of the renin-angiotensin-aldosterone system. J Am Coll Cardiol 52: 750–754. 18718423 10.1016/j.jacc.2008.02.088 PMC2856943

[25] Verma A, Shan Z, Lei B, Yuan L, Liu X, et al (2012) ACE2 and Ang-(1–7) confer protection against development of diabetic retinopathy. Mol Ther 20: 28–36. 21792177 10.1038/mt.2011.155 PMC3255596

[26] Dong B, Zhang C, Feng JB, Zhao YX, Li SY, et al (2008) Overexpression of ACE2 enhances plaque stability in a rabbit model of atherosclerosis. Arterioscler Thromb Vasc Biol 28: 1270–1276. 18403726 10.1161/ATVBAHA.108.164715

[27] Zhang C, Zhao YX, Zhang YH, Zhu L, Deng BP, et al (2010) Angiotensin-converting enzyme 2 attenuates atherosclerotic lesions by targeting vascular cells. Proc Natl Acad Sci U S A 107: 15886–15891. 20798044 10.1073/pnas.1001253107 PMC2936602

[28] Tesanovic S, Vinh A, Gaspari TA, Casley D, Widdop RE (2010) Vasoprotective and atheroprotective effects of angiotensin (1–7) in apolipoprotein E-deficient mice. Arterioscler Thromb Vasc Biol 30: 1606–1613. 20448208 10.1161/ATVBAHA.110.204453

[29] Ren J, Ceylan-Isik AF (2004) Diabetic cardiomyopathy: do women differ from men? Endocrine 25: 73–83. 15711018 10.1385/ENDO:25:2:073

[30] Sowers JR (1998) Diabetes mellitus and cardiovascular disease in women. Arch Intern Med 158: 617–621. 9521226 10.1001/archinte.158.6.617

[31] Ceylan-Isik AF, LaCour KH, Ren J (2006) Gender disparity of streptozotocin-induced intrinsic contractile dysfunction in murine ventricular myocytes: role of chronic activation of Akt. Clin Exp Pharmacol Physiol 33: 102–108. 16445707 10.1111/j.1440-1681.2006.04331.x

